# Mild hypoglycemia is independently associated with increased risk of mortality in patients with sepsis: a 3-year retrospective observational study

**DOI:** 10.1186/cc11674

**Published:** 2012-10-12

**Authors:** Sunghoon Park, Dong-Gyu Kim, Gee Young Suh, Jun Goo Kang, Young-Su Ju, Yong-Jae Lee, Ji Young Park, Seok Won Lee, Ki-Suck Jung

**Affiliations:** 1 Division of Pulmonary, Allergy and Critical Care Medicine, Hallym University Sacred Heart Hospital, Anyang 431-070, Republic of Korea; 2 Division of Pulmonary and Critical Care Medicine, Department of Medicine, Samsung Medical Center, Sungkyunkwan University School of Medicine, Seoul 135-710, Republic of Korea; 3 Division of Endocrinology and Metabolism, Hallym University Sacred Heart Hospital, Anyang 431-070, Republic of Korea; 4 Department of Occupational and Environmental Medicine, Hallym University Sacred Heart Hospital, Anyang 431-070, Republic of Korea; 5 Department of Family Medicine, Yonsei University College of Medicine, Yongin 449-930, Republic of Korea

## Abstract

**Introduction:**

Mild hypoglycemia is associated with increased mortality in critically ill patients. However, data regarding the association between mild hypoglycemia and patient outcomes among patients with sepsis are limited.

**Methods:**

Patients admitted to a medical ICU for sepsis, as defined by the Surviving Sepsis Campaign guidelines, during a 3-year period were enrolled retrospectively. Data on blood glucose (BG) control parameters and patient outcomes were collected. The primary outcome was the relationship of mild hypoglycemia (defined as minimum BG of 40 to 69 mg/dl during ICU stay) to hospital mortality, and the secondary outcomes were ICU-acquired complication rates, ICU and 1-year mortality rates. A relationship between glucose variability and hypoglycemic events was also investigated.

**Results:**

Three-hundred and thirteen consecutive patients with sepsis were enrolled (mean age, 71.8 ± 11.3 years; male, *n *= 166; diabetics, *n *= 102). A total of 14,249 (5.6/day/patient) BG tests were performed, and 175 hypoglycemic events (spontaneous, *n *= 71; iatrogenic, *n *= 104) occurred in 80 (25.6%) patients during the ICU stay; severe hypoglycemia (minimum BG level < 40 mg/dl) occurred in 24 (7.7%) patients, and mild hypoglycemia (minimum BG level 40 to 69 mg/dl) was found in 56 (17.9%) patients. The frequency of hypoglycemic events increased with higher glucose variability, and patients with mild hypoglycemia had higher rates of ICU-acquired complications than did those with no hypoglycemia (renal, 36.2% vs. 15.6%, *P *= 0.003; cardiac, 31.9% vs. 14.3%, *P *= 0.008; hepatic, 34.0% vs. 18.2%, *P *= 0.024; bacteremia, 14.9% vs. 4.5%, *P *= 0.021). Multivariate analysis revealed that mild hypoglycemia was independently associated with increased hospital mortality (odds ratio, 3.43; 95% confidence interval, 1.51 to 7.82), and even a single event was an independent risk factor (odds ratio, 2.98; 95% confidence interval, 1.10 to 8.09). Kaplan-Meier analysis demonstrated that mild hypoglycemia was significantly associated with a lower 1-year cumulative survival rate among patients with sepsis (*P *< 0.001).

**Conclusion:**

Mild hypoglycemia was associated with increased risk of hospital and 1-year mortality, as well as the occurrence of ICU-acquired complications. Physicians thus need to recognize the importance of mild hypoglycemia in patients with sepsis.

## Introduction

Since van den Berghe and colleagues demonstrated in the second Leuven study that severe hypoglycemia (blood glucose (BG) < 40 mg/dl) is associated with mortality [1], subsequent multicentre studies (Efficacy of Volume Substitution and Insulin Therapy in Severe Sepsis (VISEP) and GLUCONTROL trials) have identified a high rate of severe hypoglycemic events among intensive insulin treatment (IIT) groups (target, 80 to 110 mg/dl) [2,3]. In addition, several large observational studies have shown that severe hypoglycemia is an independent risk factor for mortality [4-6].

Several recent studies have demonstrated the relationship between mild hypoglycemia and increased mortality rate in critically ill patients. Egi and colleagues reported that patients with mild hypoglycemia had an increased unadjusted mortality rate [7], and Krinsley and colleagues also demonstrated a strong association between mild hypoglycemia and increased mortality [8]. The American Diabetes Association revised the definition of hypoglycemia as BG < 70 mg/dl in 2010 [9]. Physicians should thus be cautious of mild hypoglycemic events when treating critically ill patients.

Glucose control may be difficult in patients with sepsis because of frequent hypoglycemic and hyperglycemic events, which can have detrimental effects on hospital outcomes [10-12]. Although many glucose control studies have included patients with sepsis [2,3,7,13], the importance of hypoglycemia among patients with sepsis has been addressed by only a few studies [11,14]. In particular, data on mild hypoglycemia are limited. The primary objective of this study was therefore to investigate the association between mild hypoglycemia (BG 40 to 69 mg/dl) and hospital mortality, and the secondary objectives were to investigate its relationship with the ICU-acquired complication rate and ICU and 1-year mortality rates among patients with sepsis.

## Materials and methods

### Study population

**Figure 1:**
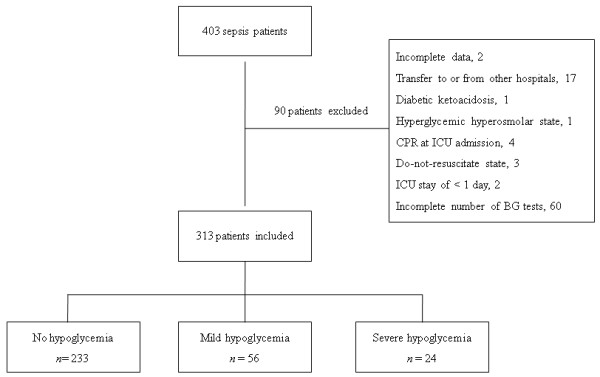
**Flow chart of patient enrolment**. BG, blood glucose; CPR, cardiopulmonary resuscitation.

Patients who were admitted to the medical ICU for sepsis at Hallym University Sacred Heart Hospital between January 2008 and December 2010 were enrolled in our study. The 2008 Surviving Sepsis Campaign guidelines were used to diagnose sepsis [15]. We collected data anonymously from electronic medical records and analyzed them retrospectively. Initially, 403 adult patients were screened for participation in the study. Patients who had daily BG tests and at least four BG tests during their ICU stay were included in the study, and 90 patients were excluded based on the exclusion criteria (Figure 1). The institutional review board at Hallym University Sacred Heart Hospital approved the present study (IRB No. 2012-I006) and waived the necessity to obtain written consent because of its retrospective nature.

### Data collection

We collected data regarding age, sex, co-morbidities, laboratory parameters, Simplified Acute Physiology Score (SAPS) II and ICU treatments (that is, insulin, any dose of systemic steroid, mechanical ventilation, renal replacement therapy). In addition to ICU-acquired complications occurring after ICU day 3, we investigated hospital, ICU, and 30-day mortalities and 1-year cumulative survival rates.

Various BG control parameters during the ICU stay were calculated for each patient as follows: frequency of BG tests (total and daily mean frequencies); mean BG levels; number of severe hypoglycemia (BG < 40 mg/dl) and mild hypoglycemia (BG 40 to 69 mg/dl) events; and three glucose variability (GV) parameters (standard deviation (SD) of mean BG levels, coefficient of variation (SD of mean BG/mean BG), and Hermanides' metric for GV) [16]

We investigated the occurrence of ICU-acquired complications occurring after ICU day 3 (that is, renal, cardiac, hepatic and respiratory complications, and bacteremia). Renal complications were defined as: an increase in serum creatinine levels of at least twofold over baseline levels; progression of oliguria (urine output < 500 ml in 24 hours or < 0.5 ml/kg/hour); or initiation of renal replacement therapy.

Cardiac complications were defined as: an ischemic event indicated by an increase in cardiac markers (troponin I and creatine kinase-MB) and significant electrocardiogram changes indicative of sustained new ischemia (ST-segment depression or elevation, new pathologic Q waves, and T-wave abnormalities in at least two consecutive leads); fatal arrhythmia (ventricular tachycardia or ventricular fibrillation); or sudden cardiac arrest.

Hepatic complications were defined as: greater than twofold increase in total bilirubin over baseline levels (and at least > 4 mg/dl); or increase in aspartate aminotransferase or alanine aminotrasferase > 1,000 IU/l with a rise in the prothrombin time (international normalized time > 2).

Respiratory complications were defined as: development of acute respiratory distress syndrome [17]; ventilator-associated pneumonia [18]; or initiation of mechanical ventilation after 3 ICU days, excluding cases of reintubation resulting from extubation failure.

Finally, bacteremia was defined as at least two positive blood culture results, excluding isolation of coagulase-negative *Staphylococcus*.

### Blood glucose control and monitoring in the ICU

In our medical ICU, BG controls were conducted using an insulin protocol (Additional file 1) that was modified according to the Surviving Sepsis Campaign guidelines [15,19]. The target for BG control was 70 to 150 mg/dl, and BG levels in arterial or capillary blood were measured using a bedside glucose meter (LifeScan, Inc., Milpitas, CA, USA). Patients received a continuous intravenous insulin infusion when BG levels were > 150 mg/dl. One unit of regular human insulin per 1 ml of 0.9% NaCl was administered, and the insulin dose was adjusted to maintain the target BG level. BG values were initially monitored every 1 to 2 hours until glucose values and insulin infusion rates were stable, and then every 4 to 6 hours thereafter.

### Data analyses

The primary outcome of the present study was the association between mild hypoglycemia and hospital mortality. The secondary outcomes included a comparison of ICU-acquired complication rates, and ICU and 30-day and 1-year mortalities between patients with and without mild hypoglycemia. Risk factors for hypoglycemic events were investigated, and the proportion of patients who experienced hypoglycemic events were also compared among the quartile groups of BG control parameters (that is, mean BG, SD, coefficient of variation, and Hermanides' metric).

Data are expressed as mean (standard deviation) or median (interquartile range) as appropriate for continuous data and as percentages for categorical data. For continuous data, Student's *t *test or the Mann-Whitney *U *test was used when comparing two groups and analysis of variance or the Kruskal-Wallis test was used when comparing three groups. The chi-squared test or Fisher's exact test was used for categorical data; however, when comparing two groups from the hypoglycemia groups (that is, mild hypoglycemia vs. no hypoglycemia; severe hypoglycemia vs. no hypoglycemia), logistic regression analysis was used in which the hypoglycemia variable was categorized into three groups. To identify risk factors for hospital mortality and hypoglycemic events, multivariate logistic regression analyses were performed using covariates with *P *< 0.05 in the univariate analyses. A backward stepwise analysis (based on the likelihood ratio) was used, with *P *= 0.05 to enter and *P *= 0.10 to stay in the model. The Kaplan-Meier method with the log-rank test was used to analyze 1-year survival rates. All reported *P *values were two-sided, and *P *< 0.05 was considered statistically significant. All analyses were conducted using SAS statistical software, EG version (SAS Institute, Inc., Cary, NC, USA).

## Results

### Study population

The mean age of the enrolled patients (*n *= 313) was 71.8 ± 11.3 years, and 166 were male (Table 1). A history of smoking was found for 74 (23.6%) patients, and diabetes (*n *= 102, 32.6%) and hypertension (*n *= 125, 39.9%) were the most common co-morbid illnesses (cancer, *n *= 63; cerebrovascular accidents, *n *= 53; chronic heart disease, *n *= 35; chronic kidney disease, *n *= 36; liver cirrhosis, *n *= 14; chronic obstructive pulmonary disease/bronchial asthma, *n *= 25).

**Table 1:** Glucose control parameters, treatments, and outcomes among enrolled patients stratified by minimum blood glucose levels

	Minimum blood glucose level			***P *value**^**a**^
		
	< 40 mg/dl (*n *= 24)	40 to 69 mg/dl (*n *= 56)	≥ **70 mg/dl (*n *= 233)**	
Age	71.9 ± 11.2	69.3 ± 12.6	72.4 ± 11.0	0.177^b^
Male/female	11/13	34/22	121/112	0.379^c^
Diabetes	10 (41.7%)	31 (55.4%)	61 (26.2%)	< 0.001^c^
Admission SAPS II	57 ± 15.9	52.2 ± 14.0	46.4 ± 13.1	< 0.001^b^
Laboratory parameters				
WBC (mm^3^)	11,550 (6,950 to 20,775)	13,450 (6,225 to 20,075)	14,300 (8,850 to 21,050)	0.053^d^
Hematocrit (%)	32.2 ± 6.0	31.1 ± 7.8	34.8 ± 6.7	0.001^b^
Platelet (×10^3 ^mm^3^)	166.0 (73.8 to 268.3)	190.0 (101.0 to 288.3)	209.0 (121.0 to 288.0)	0.320^d^
Lactate (mmol/l)	4.8 (3.5 to 8.6)	4.5 (2.6 to 7.0)	3.5 (2.3 to 5.6)	0.013^d^
Serum albumin (g/dl)	2.9 ± 0.6	3.0 ± 0.6	3.2 ± 0.6	0.020^b^
Blood urea nitrogen (mg/dl)	28.3 (17.8 to 41.3)	36.6 (18.1 to 50.7)	25.6 (16.4 to 42.3)	0.237^d^
Creatinine (mg/dl)	1.1 (0.7 to 2.5)	1.3 (0.9 to 2.3)	1.2 (0.8 to 1.9)	0.345^d^
Total bilirubin (mg/dl)	0.9 (0.6 to 1.4)	0.9 (0.5 to 1.7)	0.9 (0.6 to 1.9)	0.744^d^
BNP (pg/ml)	554.4 (207.3 to 1205.4)	347.3 (133.8 to 1377.4)	251.9 (138.8 to 638.2)	0.029^d^
CK-MB (ng/ml)	3.9 (2.0 to 7.9)	2.3 (1.3 to 8.2)	2.0 (0.9 to 4.95)	0.023^d^
C-reactive protein (mg/l)	170.9 (77.3 to 232.1)	142.0 (86.9 to 225.0)	157.0 (81.6 to 239.5)	0.931^d^
BG control parameters				
Number of BG tests (/day/patient)	6.3 (4.9 to 9.9)	6.4 (5.0 to 7.9)	5.2 (4.3 to 6.7)	< 0.001^d^
Mean BG (mg/dl)^e^	163.9 (146.0 to 194.3)	189.3 (166.9 to 208.1)	174.6 (145.0 to 205.9)	0.096^d^
Median SD (mg/dl)	73.1 (48.9 to 116.2)	71.3 (46.1 to 92.9)	42.1 (30.5 to 62.0)	< 0.001^d^
Median CV (%)	41.6 (29.4 to 56.5)	37.6 (29.2 to 45.2)	24.2 (18.9 to 31.8)	< 0.001^d^
Hermanides' metric (mg/dl)	11.9 (6.9 to 22.1)	11.7 (8.0 to 16.0)	6.6 (4.5 to 9.6)	< 0.001^d^
Insulin therapy	18 (75.0%)	43 (76.8%)	142 (60.9%)	0.015^c, f^
Systemic steroid therapy	9 (37.5%)	26 (46.4%)	91 (39.1%)	0.576^c, f^
Vasopressors	23 (95.8%)	45 (80.4%)	166 (71.2%)	0.017^c, f^
Mechanical ventilation	23 (95.8%)	40 (71.4%)	132 (56.7%)	< 0.001^c, f^
VAP	3 (12.5%)	6 (10.7%)	7 (3.0%)	0.015^c, f^
Renal replacement therapy	5 (20.8%)	11 (19.6%)	25 (10.7%)	0.150^c^
Length of ICU stay (days)	11.5 (3.3-19.8)	7.0 (5.0-14.0)	5.0 (3.0-9.0)	< 0.001^d^
ICU mortality	18/24 (75.0%)	34/56 (60.7%)	66/233 (28.3%)	< 0.001^c, f^
Hospital mortality	19/24 (79.2%)	37/56 (66.1%)	71/233 (30.5%)	< 0.001^c, f^
30-day mortality	18/24 (75.0%)	34/55 (61.8%)	69/230 (30.0%)	< 0.001^c, f^
1-year mortality	21/24 (87.5%)	41/55 (74.5%)	107/230 (46.5%)	< 0.001^c, f^

Continuous data expressed as mean ± standard deviation or median (interquartile range) and categorical data expressed as number (%). BG, blood glucose; BNP, brain natriuretic peptide; CK-MB, creatine kinase-MB; CV, coefficient of variation; SAPS, Simplified Acute Physiology Score; SD, standard deviation; VAP, ventilator-associated pneumonia; WBC, white blood cell. ^a^Comparisons among three groups (that is, < 40 mg/dl vs. 40 to 69 mg/dl vs. ≥ 70 mg/dl). ^b^Analysis of variance. ^c^Chi-squared test. ^d^Kruskal-Wallis test. ^e^Median values of individual mean BG levels. ^f^*P *< 0.05 by linear-by-linear association.

Septic shock accounted for 59.4% of the patients (*n *= 186; severe sepsis, *n *= 99; sepsis, *n *= 28), and among the sepsis origins pneumonia (*n *= 171, 54.6%) was the most common (urinary tract, *n *= 58; hepatobiliary, *n *= 55; intra-abdominal, *n *= 14; soft tissue and skin, *n *= 8; bacteremia, *n *= 3; meningitis, *n *= 2; unidentified, *n *= 2). Insulin therapy was conducted in 83 (81.4%) diabetics (vs. 120 (56.9%) nondiabetics).

### Blood glucose measurements, hypoglycemic events, and risk factors

The total number of BG tests performed in all enrolled patients during their ICU stay was 14,249, and the median (interquartile range) number of BG tests per day per patient was 5.6 (4.4 to 7.0). The median value of individual mean BG levels was 177.3 mg/dl (148.8 to 205.6 mg/dl). The median values for the SD, coefficient of variation, and Hermanides' metric were 48.4 mg/dl (33.6 to 71.0 mg/dl), 27.6% (20.0 to 36.5%), and 7.4 mg/dl (4.9 to 11.8 mg/dl), respectively. In total, 175 hypoglycemic events (BG < 70 mg/dl) occurred in 80 (25.6%) patients. Among these, 71 (40.6%) hypoglycemic events occurred spontaneously and 104 (59.4%) occurred with insulin therapy (that is, iatrogenic hypoglycemia). A total of 83 (47.4%) events occurred in conscious patients and 92 (52.6%) events occurred in unconscious patients. Among the 80 hypoglycemic patients, 56 had a minimum BG level of 40 to 69 mg/dl (that is, mild hypoglycemia) and 24 had a minimum BG level < 40 mg/dl (that is, severe hypoglycemia); 13 patients had both mild and severe hypoglycemia during their ICU stay.

**Figure 2:**
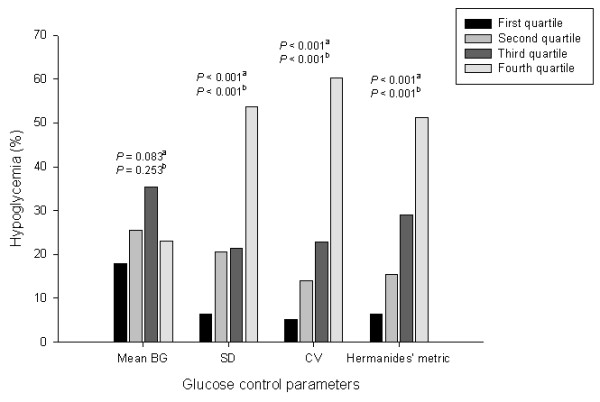
**Frequencies of hypoglycemia during ICU stay stratified by quartiles of glucose control parameters**. Quartile ranges of mean blood glucose (BG): < 148.8, 148.8 to 177.3, 177.3 to 205.6, and > 205.6 mg/dl. Quartile ranges of standard deviation (SD): < 33.6, 33.6 to 48.4, 48.4 to 71.0, and > 71.0 mg/dl. Quartile ranges of coefficient of variation (CV): < 20.0%, 20.0 to 27.6%, 27.6 to 36.5%, and > 36.5%. Quartile ranges of Hermanides' metric: < 4.9, 4.9 to 7.4, 7.4 to 11.8, and > 11.8 mg/dl. ^a^*P *< 0.001 (chi-squared test), ^b^*P *< 0.001 (linear-by-linear association) among the quartile groups.

When the patients were stratified based on their minimum BG levels, the frequency of diabetes and insulin treatment, SAPS II, SD of the mean BG, coefficient of variation, and Hermanides' metric were higher in the two hypoglycemic groups as compared with patients without hypoglycemia (Table 1). The frequency of hypoglycemic events tended to increase in patients with higher GV parameters (Figure 2).

The univariate analyses revealed 16 variables - diabetes, hematocrit, lactate, serum albumin, brain natriuretic peptide, creatine kinase-MB, GV parameters (SD of mean BG, coefficient of variation, Hermanides' metric), SAPS II, insulin therapy, vasopressor, mechanical ventilation, renal replacement therapy, hepatic failure, and ventilator-associated pneumonia - that were significantly associated with hypoglycemic events (*P *< 0.05). Multivariate logistic analysis included 14 variables; of the three GV parameters, only the SD of mean BG was included in the model. Among the variables included in the final model, diabetes, hematocrit, lactate, SD of mean BG, hepatic failure, and ventilator-associated pneumonia were independent risk factors for hypoglycemic events (Table 2).

**Table 2:** Multivariate analysis of risk factors for hypoglycemic events

Variable	Odds ratio	95% confidence interval	*P *value
Diabetes	2.60	1.30 to 5.17	0.007
Hematocrit (%)	0.91	0.87 to 0.96	0.001
Lactate (mmol/l)	1.11	1.02 to 1.21	0.017
CK-MB (ng/ml)	1.02	1.00 to 1.05	0.050
SD of mean BG	1.03	1.02 to 1.04	< 0.001
Hepatic failure	3.63	1.52 to 8.67	0.004
VAP	11.39	3.43 to 37.85	< 0.001

BG, blood glucose; CK-MB, creatine kinase-MB; SD, standard deviation; VAP, ventilator-associated pneumonia. Chi-square = 5.336 and *P *= 0.721 by Hosmer-Lemeshow goodness-of-fit test.

### Mild hypoglycemic events and the occurrence of ICU-acquired complications

**Figure 3:**
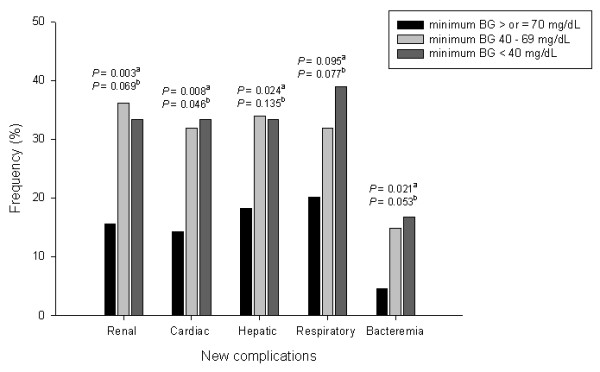
**Frequency of ICU-acquired complications in patients with ICU stay > 3 days**. Data stratified by minimum blood glucose (BG) levels. The frequencies of renal, cardiac, and hepatic complications and bacteremia were significantly higher in patients with mild hypoglycemia as compared with those without hypoglycemia. ^a^Comparisons between patients with mild hypoglycemia and those without hypoglycemia. ^b^Comparisons between patients with severe hypoglycemia and those without hypoglycemia.

We investigated the occurrence of ICU-acquired complications among 219 patients who stayed in the ICU for more than 3 days, and 202 complications occurred in 116 (53%) patients (renal, *n *= 47; cardiac, *n *= 35; hepatic, *n *= 50; respiratory, *n *= 53; bacteriemia, *n *= 17; Figure 3). The complication rates were higher in both hypoglycemic groups (mild hypoglycemia, 33/47; severe hypoglycemia, 15/18) as compared with patients without hypoglycemia (68/154; *P *= 0.002 and *P *= 0.005, respectively). The frequencies of renal, cardiac, and hepatic complications and bacteremia were higher in patients with mild hypoglycemia as compared with those who were not hypoglycemic (36.2% vs. 15.6%, *P *= 0.003; 31.9% vs. 14.3%, *P *= 0.008; 34.0% vs. 18.2%, *P *= 0.024; and 14.9% vs. 4.5%, *P *= 0.021, respectively). Among 48 patients with both hypoglycemia and ICU-acquired complications, hypoglycemic events occurred prior to ICU-acquired complications in 31 patients.

### Mild hypoglycemia and mortality

The ICU, hospital, 30-day, and 1-year mortalities were higher in patients with mild hypoglycemia compared with those without hypoglycemia. We found an increasing trend of mortality in patients with increasing severity of hypoglycemia (Table 1). Furthermore, we observed a trend of increasing mortality in patients with the highest frequency (0 vs. 1 to 2 vs. ≥ 3) of hypoglycemia events, irrespective of whether the hypoglycemia was mild or severe (Additional file 2). We also found no significant differences between spontaneous and iatrogenic hypoglycemia in terms of the time from ICU admission to hypoglycemic event, time from hypoglycemic event to hospital death, and hospital mortality rate (Table 3).

**Table 3:** Comparisons of time to events and time to hospital deaths among three hypoglycemic groups

Variable	Spontaneous hypoglycemia (patient *n *= 32)	Iatrogenic hypoglycemia (patient *n *= 41)	Both hypoglycemias (patient *n *= 7)	***P *value**^**a**^
Total number of hypoglycemic events	61	81	33^b^	
Hospital deaths	22 (68.8%)	28 (68.3%)	6 (85.7%)	0.967^c^
Time from ICU admission to first hypoglycemic event (days)	3.0 (1.0 to 5.8)	3.0 (1.0 to 5.5)	1.0 (6 hours to 4.0)	0.490^d^
Time from first hypoglycemic event to hospital death (days)	3.0 (1.0 to 12.0)	5.0 (1.0 to 11.5)	13.5 (6.0 to 26.5)	0.898^d^

^a^Comparisons between two groups (that is, spontaneous hypoglycemia vs. iatrogenic hypoglycemia). ^b^Ten hypoglycemic events occurred spontaneously. ^c^Chi-squared test. ^d^Mann-Whitney *U *test.

In the multivariate analysis, 16 variables significant at *P *< 0.05 in the univariate analyses (Additional file 3) were initially included. Among the three GV parameters, the SD of the mean BG was the only variable included in the model, and the hypoglycemia variable was categorized into three groups based on minimum BG levels. In the final model (Table 4), mild hypoglycemia was significantly associated with increased hospital mortality (odds ratio, 3.43; 95% confidence interval, 1.51 to 7.82). We performed a further multivariate analysis in patients who experienced no or one mild hypoglycemic event (*n *= 233 and 32, respectively; Additional file 4) and determined that a single mild hypoglycemic event was significantly associated with increased hospital mortality (OR, 2.98; 95% confidence interval, 1.10 to 8.09).

**Table 4:** Multivariate analysis of risk factors for hospital mortality (*n *= 313)

Variable	Odds ratio	95% confidence interval	*P *value
Cancer	2.82	1.24 to 6.41	0.014
Serum albumin (mg/dl)	0.34	0.19 to 0.62	< 0.001
Admission SAPS II	1.03	1.00 to 1.06	0.027
Vasopressors	2.15	0.85 to 5.41	0.105
Mechanical ventilation	3.56	1.61 to 7.85	0.002
Hepatic failure	18.27	3.90 to 85.61	< 0.001
VAP	11.33	2.03 to 63.30	0.006
Hypoglycemic events			0.009
Mild hypoglycemia	3.43	1.51 to 7.82	0.003
Severe hypoglycemia	2.17	0.59 to 7.94	0.244

SAPS, Simplified Acute Physiology Score; VAP: ventilator-associated pneumonia. Chi-square = 4.826 and *P *= 0.776 by Hosmer-Lemeshow goodness-of-fit test.

**Figure 4:**
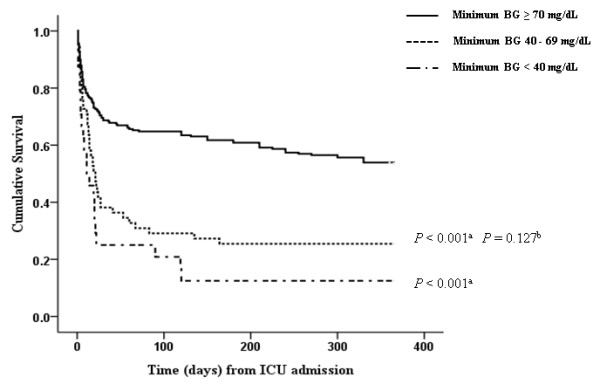
**Kaplan-Meier survival analyses for 1-year cumulative survival rates**. Among the three hypoglycemia groups, patients with mild hypoglycemia had a significantly lower 1-year survival rate than did patients without hypoglycemia. BG, blood glucose. ^a^*P *< 0.001, ^b^*P *= 0.127 vs. severe hypoglycemia.

The overall survival rate for all enrolled patients was 45.3% (140/309) at 1 year; four patients were lost to the 1-year follow-up. Mean survival times were 225.8 ± 10.9 days, 111.1 ± 20.4 days, and 66.6 ± 24.0 days in patients with no hypoglycemia, mild hypoglycemia, and severe hypoglycemia, respectively (Figure 4). The 1-year survival rates were significantly lower in the mild and severe hypoglycemic groups compared with patients without hypoglycemia, and no significant difference was observed between the two hypoglycemia groups.

## Discussion

The present study identified several important findings in patients with sepsis. First, mild hypoglycemia (defined as BG 40 to 69 mg/dl) was significantly associated with increased hospital mortality, and even a single mild hypoglycemic event showed a significant association. Second, mild hypoglycemia was significantly associated with a lower 1-year cumulative survival rate. Third, mild hypoglycemia was associated with a high rate of ICU-acquired complications during the ICU stay.

Glucose control in patients with sepsis can be challenging because they are vulnerable to hyperglycemia and hypoglycemia, which result in high GV [10-12]. In the present study, the median value of individual mean BG levels (177.3 mg/dl (148.8 to 205.6 mg/dl)) suggested that BG was not well controlled, and insulin treatment was inadequate in patients with sepsis. However, the rate of hypoglycemia was high. In a study by Bagshaw and colleagues, the incidence of mild hypoglycemia was 6.2% in all patients and 8.6% among the sepsis subgroup [4]. The authors reported that the adjusted odds ratio for hospital mortality was 1.6 (95% confidence interval, 1.4 to 1.8). Egi and colleagues [7] and Krinsley and colleagues [8], more specifically, identified that mild hypoglycemia had a greater than twofold increase in the odds of hospital mortality. These previous studies targeted all ICU patients. However, we focused on patients with sepsis and noticed that even a single mild hypoglycemic event was associated with a nearly threefold increase in hospital mortality. In particular, when we stratified sepsis patients by tertile groups of SAPS II, patients with mild hypoglycemia showed a higher mortality rate than those without hypoglycemia in each tertile group (Additional file 5). This finding suggests that mild hypoglycemia *per se *is associated with patient outcomes independent of disease severity. However, the multivariate analysis did not reveal a significant association between severe hypoglycemia and mortality. We believe that the small number of patients with severe hypoglycemia in our study may, in part, account for this finding. A future study with a larger number of patients is thus needed to clarify this point.

We demonstrated that the long-term survival rate was significantly lower in patients with sepsis who experienced mild hypoglycemia as compared with patients with sepsis who were not hypoglycemic (Figure 4). Although the authors of the Normoglycaemia in Intensive Care Evaluation - Surviving Using Glucose Algorithm Regulation (NICE-SUGAR) trial noticed that severe hypoglycemia was more frequent and that 90-day mortality was higher in the IIT group [13], the long-term prognosis of patients with hypoglycemia has not been well studied. The NICE-SUGAR trial found no difference between the IIT and conventional-therapy groups in rates of new organ failure; however, death from cardiovascular causes was more common in the IIT group. The present study found that mild hypoglycemia was associated with increased rates of renal, cardiac, and hepatic complications and bacteremia. Although the definitions for the ICU-acquired complications in the present study were different from those used in the VISEP and NICE-SUGAR trials [2,13], our results are additive to those of previous studies.

GV parameters are associated with hypoglycemia and mortality [10,20]. The results of the present study show that the SD of mean BG levels, one of three GV parameters, was a significant risk factor for hypoglycemia. GV-reduction strategies may thus be critical for decreasing hypoglycemic events and hospital mortality [20]. We also identified other risk factors for hypoglycemia such as diabetes, hematocrit, lactate, hepatic failure, and ventilator-associated pneumonia; however, previous studies have found renal failure, renal replacement therapy, and the use of vasopressors to be risk factors [6,10]. Although physicians must take care in the management of patients with these risk factors, it is important that they recognize that hypoglycemia may simply represent a marker of worsening organ function [21].

Several factors may explain the relationship between hypoglycemia and patient outcomes [21]. During critical illnesses, the physiological responses induced by hypoglycemia, such as the inhibition of insulin release and increased release of glucagon, epinephrine, growth hormone, and cortisol, are frequently impaired [22,23]. Second, the large amount of glucose administered to patients with hypoglycemia may be associated with cellular damage [24]. Third, hypoglycemia may cause biological toxicities, such as an increased systemic inflammatory response [25], cerebral vasodilation [26], and neuroglycopenia [27]. In particular, severe hypoglycemia causes an increase in extracellular glutamate and results in excitotoxicity [28]. However, as mentioned previously, hypoglycemia may be a marker of increasing disease severity or impending death [21]. Although we demonstrated that mild hypoglycemia was an independent risk factor for hospital mortality, the causal relationship is not clear.

The present study had several limitations. First, it was a retrospective study, and the number of patients was limited. The data may thus reflect unintended bias. Second, capillary blood sampling was performed in a percentage of patients using a bedside glucose analyzer, which may be associated with analytic inaccuracies in critically ill patients [29-31]. However, this measurement is typically used in the ICU setting. Third, BG control in this investigation was challenging, reflected by the fact that the lowest quartile of the mean BG was < 148.8 mg/dl, while the glycemic target was < 150 mg/dl. Fourth, although we analyzed the association between the frequency of hypoglycemia and mortality, intermittent monitoring of BG in the present study may have led to undetected hypoglycemic events. Fifth, we could not investigate the insulin dosage or nutritional support (that is, calorie intake), which can impact hypoglycemic events and patient outcomes.

Despite these limitations, our study has several major strengths. First, in contrast to previous studies, we focused on mild hypoglycemic events in patients with sepsis. Although the causal relationship is unclear, a single mild hypoglycemic event may be harmful to patients. Second, we performed various analyses to elucidate the relationship between mild hypoglycemia and patient outcome. In particular, although this is a retrospective study, we obtained 1-year survival data for most of the patients (98.7%), which we think provide credible evidence regarding long-term outcome. Future large-scale, prospective studies of patients with hypoglycemia are necessary to clarify the relationship between mild hypoglycemia and patient outcome. Furthermore, consideration of hypoglycemic conditions (spontaneous vs. iatrogenic hypoglycemia) and diabetic status is also likely to yield intriguing results.

## Conclusion

Mild hypoglycemia was associated with increased hospital and 1-year mortality in patients with sepsis. Furthermore, it was also associated with an increased rate of ICU-acquired complications. Physicians must thus be aware of the importance of mild hypoglycemia in patients with sepsis.

## Key messages

• Mild hypoglycemia, defined as a BG of 40 to 69 mg/dl, was significantly associated with increased hospital mortality, and even a single mild hypoglycemic event was an independent risk factor.

• Mild hypoglycemia was significantly associated with a lower 1-year cumulative survival rate among patients with sepsis.

• Mild hypoglycemia was associated with a high rate of ICU-acquired complications during the ICU stay in patients with sepsis.

## Abbreviations

BG: blood glucose; GV: glucose variability; IIT: intensive insulin treatment (target, 80 to 110 mg/dl); NICE-SUGAR: Normoglycemia in Intensive Care Evaluation - Surviving Using Glucose Algorithm Regulation; SAPS: Simplified Acute Physiology Score; SD: standard deviation; VISEP: Efficacy of Volume Substitution and Insulin Therapy in Severe Sepsis.

## Competing interests

The authors declare that they have no competing interests.

## Authors' contributions

SP and K-SJ contributed to the design of the study and drafted the manuscript. D-GK, GYS, JGK, Y-JL, JYP, and SWL contributed to data analysis and interpretation of the results. Y-SJ contributed to statistical analysis. All authors read and approved the final manuscript.

## Supplementary Material

Additional file 1 **a diagram showing the glycemic control protocol (Hallym University Sacred Heart Hospital)**. Click here for file

Additional file 2 **a figure showing the associations between the number of hypoglycemic events (0 vs. 1 to 2 vs. ≥ 3 hypoglycemia events) and hospital mortality**. Click here for file

Additional file 3 **a table presenting the univariate analyses of risk factors for hospital mortality**. Click here for file

Additional file 4 **a table presenting the multivariate analysis of risk factors for hospital mortality among patients with 0 or 1 episode of mild hypoglycemia**. Click here for file

Additional file 5 **a figure showing the relationship between hypoglycemia and hospital mortality, stratified by SAPS II tertiles**. Click here for file
