# Supplementary material for: Mild hypoglycemia is independently associated with increased risk of mortality in patients with sepsis: a 3-year retrospective observational study
Source: Crit Care. 2012 Oct 12;16(5):R189. doi: 10.1186/cc11674 (PMC3682291; doi:10.1186/cc11674)
Supplement: Additional file 1 — a diagram showing the glycemic control protocol (Hallym University Sacred Heart Hospital). [file cc11674-S1.DOC]

**PROTOCOL FOR GLUCOSE CONTROL IN SEPSIS**

Hallym university medical center
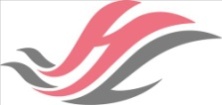


**TARGET BLOOD GLUCOSE LEVEL : 70 - 150 mg/dL**

General Information

- Start a continuous intravenous (IV) insulin infusion when BG levels are > 150 mg/dL.
- One unit of regular insulin per 1cc 0.9% NaCl is administered, and the insulin dose is then adjusted to maintain the target level.
- All patients receiving IV insulin should receive a glucose calorie source
- BG values are initially monitored every 1-2 h until glucose values and insulin infusion rates are stable, and then every 4-6 h thereafter.
- Low glucose levels obtained with point-of-care testing of capillary blood must be interpreted with caution because such measurements may overestimate arterial blood or plasma glucose levels.

**Starting infusion**

| **BG** | **Insulin infusion rate** |
| --- | --- |
| < 150 mg/dL | On hold |
| 150-250 mg/dL | 1 unit/h |
| 250-300 mg/dL | 2 unit/h |
| > 300 mg/dL | 4 unit/h |

**Maintenance infusion**

| **BG** | **Incremental Insulin infusion rate** |
| --- | --- |
| > 300 mg/dL | +3 unit/h |
| 250-300 mg/dL | +2 unit/h |
| 150-250 mg/dL | +1 unit/h |
| 70-150 mg/dL | + 0 unit/h (target range) |
| 40-70 mg/dL | Stop insulin,  Follow the treatment protocol for hypoglycemia |
| < 40 mg/dL | Stop insulin, Call physician immediately,  Follow the treatment protocol for hypoglycemia |

**TREATMENT PROTOCOL FOR HYPOGLYCEMIA**

Hallym university medical center
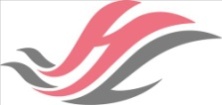


General Information

- Check fingerstick blood glucose (BG) if patient experiences any symptoms of hypoglycemia including Pallor, Clammy skin, Hunger, Restless sleep, Fatigue, Headache, Confusion, Sweatiness and Irritability
- If bedside BG < 70, confirm by repeating bedside test immediately, and *Do Not Delay Hypoglycemia Treatment*
- If the patient is taking oral medication for diabetes in the sulfonylurea class then holds the sulfonylurea, follow this algorithm.

CONSCIOUS PATIENT

ABLE to SWALLOW

UNCONSCIOUS PATIENT and or

UNABLE to SWALLOW

1. Obtain Vital sign & BG

2. If BG is 40-69 mg/dL

Treat immediately

Give ONE 15 g Carbohydrate

120 cc fruit juice

120 cc soda pop

240 cc skim milk

3. If BG is below 40 mg/dL

Treat immediately, Notify physician

Give ONE 30 g Carbohydrate

240 cc fruit juice

240 cc soda pop

4. Recheck BG in 15min

1. Obtain Vital sign & BG

2. If BG is 40-69 mg/dL

Treat immediately

Give 20 cc of 50% Dextrose IV, and

Start 5% Dextrose at 100 mL/h

3. If BG is below 40 mg/dL

Treat immediately, Notify physician

Give 50 cc of 50% Dextrose IV, and

Start 5% Dextrose at 100 mL/h

4. If unable to obtain IV access

Glucagon 1mg IM

5. Recheck BG in 15min

1. If BG < 70 at 15 minutes, then re-treat and notify doctor.
2. Document BG 15 minutes after second treatment
