# Supplementary material for: Mild hypoglycemia is independently associated with increased risk of mortality in patients with sepsis: a 3-year retrospective observational study
Source: Crit Care. 2012 Oct 12;16(5):R189. doi: 10.1186/cc11674 (PMC3682291; doi:10.1186/cc11674)
Supplement: Additional file 2 — a figure showing the associations between the number of hypoglycemic events (0 vs. 1 to 2 vs. ≥ 3 hypoglycemia events) and hospital mortality. [file cc11674-S2.DOC]

**Additional file 2**

**Title:** **Associations between the number of hypoglycemic events (0 vs. 1–2 vs. > or = 3 hypoglycemia events) and hospital mortality.** ^a^*P* < 0.001 by Chi-squared test and ^b^*P* < 0.001 by linear-by-linear association among the three groups.
