# Supplementary material for: Mild hypoglycemia is independently associated with increased risk of mortality in patients with sepsis: a 3-year retrospective observational study
Source: Crit Care. 2012 Oct 12;16(5):R189. doi: 10.1186/cc11674 (PMC3682291; doi:10.1186/cc11674)
Supplement: Additional file 3 — a table presenting the univariate analyses of risk factors for hospital mortality. [file cc11674-S3.DOC]

**Additional file 3**

**Title: Univariate analyses of risk factors for hospital mortality**

| Variables | Survivors  (n = 186) | Non-survivors  (n = 127) | OR | *P* value |
| --- | --- | --- | --- | --- |
| Age (years) | 72.2 ± 11.0 | 71.4 ± 11.8 | 0.99 | 0.542 |
| Male/female | 89/97 | 77/50 | 0.60 | 0.027 |
| Smoking | 41 (22.0%) | 33 (26.0%) | 1.24 | 0.421 |
| Alcohol intake | 29 (15.6%) | 25 (19.7%) | 1.33 | 0.348 |
| Co-morbid illnesses |  |  |  |  |
| Diabetes | 67 (36.0%) | 35 (27.6%) | 0.68 | 0.118 |
| Hypertension | 82 (44.1%) | 43 (33.9%) | 0.65 | 0.070 |
| COPD/BA | 18 (9.7%) | 7 (5.5%) | 0.54 | 0.187 |
| Liver cirrhosis | 11 (5.9%) | 3 (2.4%) | 0.39 | 0.149 |
| Chronic kidney disease | 16 (8.6%) | 20 (15.7%) | 1.99 | 0.055 |
| Chronic heart disease | 23 (12.4%) | 12 (9.4%) | 0.74 | 0.423 |
| Cerebrovascular accidents | 36 (19.4%) | 17 (13.4%) | 0.64 | 0.169 |
| Cancer | 26 (14.0%) | 37 (29.1%) | 2.53 | 0.001 |
| Laboratory parameters |  |  |  |  |
| WBC (mm^3^) | 14,950 (10,125-21,325) | 13,200 (5,500-20,700) | 0.97 | 0.027 |
| Hematocrit (%) | 35.0 ± 6.8 | 32.4 ± 7.1 | 0.95 | 0.002 |
| Platelet (mm^3^) | 211.0K (123.5K-277.5K) | 188.0K (94.0K-289.0K) | 0.64 | 1.000 |
| Lactate (mmol/L) | 3.7 (2.3-5.5) | 3.7 (2.6-6.9) | 1.04 | 0.176 |
| Serum albumin (g/dL) | 3.3 ± 0.6 | 2.9 ± 0.6 | 0.38 | <0.001 |
| Blood urea nitrogen (mg/dL) | 23.9 (15.9-44.3) | 30.4 (18.0-42.6) | 1.02 | 0.421 |
| Creatinine (mg/dL) | 1.2 (0.8-2.0) | 1.2 (0.8-2.1) | 0.98 | 0.730 |
| Total bilirubin (mg/dL) | 0.9 (0.6-1.9) | 0.8 (0.5-1.5) | 0.92 | 0.346 |
| BNP (pg/mL) | 219.3 (131.4-586.8) | 348.5 (151.0-919.9) | 1.00 | 0.036 |
| CK-MB (ng/mL) | 1.8 (0.8-3.9) | 3.4 (1.5-8.0) | 1.02 | 1.015 |
| C-reactive protein (mg/L) | 157.5 (79.8-240.8) | 148.0 (94.1-228.3) | 1.00 | 0.645 |
| Glucose control parameters |  |  |  |  |
| Mean BG (mg/dL)^a^ | 174.1 (144.5 – 204.8) | 180.3 (154.4 – 208.1) | 1.01 | 0.059 |
| Median SD (mg/dL) | 42.5 (30.4 – 64.4) | 56.3 (39.9 – 81.6) | 1.02 | <0.001 |
| Median CV (%) | 25.1 (18.9 – 33.3) | 29.4 (23.0 – 40.6) | 1.04 | <0.001 |
| Hermanides’ metric (mg/dL) | 7.1 (4.8 – 10.8) | 8.1 (5.3 – 13.2) | 1.07 | 0.001 |
| Hypoglycemia | 24 (12.9%) | 56 (44.1) | 5.34 | <0.001 |
| Admission SAPS II | 43.4 ± 11.9 | 55.2 ± 13.6 | 1.08 | <0.001 |
| Insulin therapy | 110 (59.1%) | 93 (73.2%) | 1.89 | 0.011 |
| Steroid therapy | 57 (30.6%) | 69 (54.3%) | 2.69 | <0.001 |
| Vasopressors | 118 (63.4%) | 116 (91.3%) | 6.08 | <0.001 |
| Mechanical ventilation | 83 (44.6%) | 112 (88.2%) | 9.27 | <0.001 |
| Renal replacement therapy | 14 (7.5%) | 27 (21.3%) | 3.32 | 0.001 |
| Hepatic failure | 2 (1.1%) | 35 (27.6%) | 35.00 | < 0.001 |
| VAP | 2 (1.1%) | 14 (11.0%) | 11.40 | 0.001 |

Continuous data are expressed as mean ± standard deviation or median (interquartile range) and categorical data are expressed as number (%). BA: bronchial asthma; BG: blood glucose; BNP: brain natriuretic peptide; CK-MB: creatine kinase-MB; COPD: chronic obstructive pulmonary disease; CV: coefficient of variation; ICU: intensive care unit; OR, odds ratio; SAPS II: simplified acute physiology score II; SD: standard deviation; VAP: ventilator-associated pneumonia; WBC: white blood cell. ^a^Median value of individual mean BG levels.
