# Supplementary material for: Mild hypoglycemia is independently associated with increased risk of mortality in patients with sepsis: a 3-year retrospective observational study
Source: Crit Care. 2012 Oct 12;16(5):R189. doi: 10.1186/cc11674 (PMC3682291; doi:10.1186/cc11674)
Supplement: Additional file 4 — a table presenting the multivariate analysis of risk factors for hospital mortality among patients with 0 or 1 episode of mild hypoglycemia. [file cc11674-S4.DOC]

**Additional file 4**

**Title: Multivariate analysis of risk factors for hospital mortality among patients with 0 or 1 episode of mild hypoglycemia^a^**

| Variables | Odds ratio | 95% confidence interval | *P* value |
| --- | --- | --- | --- |
| Cancer | 2.61 | 1.10 – 6.19 | 0.029 |
| Serum albumin (g/dL) | 0.34 | 0.18 – 0.63 | 0.001 |
| Admission SAPS II | 1.05 | 1.01 – 1.08 | 0.012 |
| Systemic steroid therapy | 1.87 | 0.95 – 3.68 | 0.072 |
| Mechanical ventilation | 3.50 | 1.57 – 7.84 | 0.002 |
| Hepatic failure | 9.50 | 1.91 – 47.26 | 0.006 |
| VAP | 9.28 | 1.53 – 56.19 | 0.015 |
| Mild hypoglycemia | 2.98 | 1.10 – 8.09 | 0.032 |

**^a^**Sixteen variables with *P* value of < 0.05 (ie., gender, mild hypoglycemia, steroid therapy, insulin therapy, mechanical ventilation, vasopressors, cancer, hematocrit, mean BG [blood glucose], standard deviation of mean BG, serum albumin, admission SAPS II, creatine kinase MB [CK-MB], renal replacement therapy, hepatic failure, and ventilator-associated pneumonia [VAP]) were initially included (Hosmer–Lemeshow goodness-of-fit test; Chi square = 8.215 and *P* = 0.413). ICU: intensive care unit; SAPS II: simplified acute physiology score II.
