# Supplementary material for: Mild hypoglycemia is independently associated with increased risk of mortality in patients with sepsis: a 3-year retrospective observational study
Source: Crit Care. 2012 Oct 12;16(5):R189. doi: 10.1186/cc11674 (PMC3682291; doi:10.1186/cc11674)
Supplement: Additional file 5 — a figure showing the relationship between hypoglycemia and hospital mortality, stratified by SAPS II tertiles. [file cc11674-S5.DOC]

**Additional file 5**

**Title: Relationship between hypoglycemia and hospital mortality, stratified by SAPS II tertiles (< 42.0, 42.0 to 53.0, and > 53.0).** There was a significant association between mild hypoglycemia and hospital mortality in each of tertile groups of SAPS II. ^a^Mild hypoglycemia vs. no hypoglycemia. ^b^Severe hypoglycemia vs. no hypoglycemia. SAPS II: simplified acute physiology score II.
